# Angina Pectoris*Read before the Buffalo Medical Club, Nov. 9, 1881.

**Published:** 1881-12

**Authors:** H. R. Hopkins


### BUFFALO

### Medical and Surgical Journal.

Vol. XXI. DECEMBER, 1881. No. 5.

### Original Communications.

### Angina Pectoris.*

By H. R. Hopkins, M. D.

What is angina pectoris? To answer this question with such scientific accuracy, as would meet the approval of your Committee on Pathology, would require more exact information than I have been able to glean from the literature of the subject that has fallen under my observation. The reason of this is found in the inherent difficulties of the subject, and not because the disease is one which has attracted little attention. For in the whole round of death-producing maladies, there is none that attacks its victims with more terrible and deadly energy; none, in which the picture of "death struck short on life" is more often seen, and none whose victims are more eminent and widely known. Hence it is that investigations into the nature and cause of angina pectoris have not been wanting, and its students have been among the leaders of our profession, have been men who availed themselves of every advantage that the science of medicine could offer, and as a result of their labor, we have several more or less plausible hypotheses, but we are still far from a settlement of the question.

* Read before the Buffalo Medical Club, Nov. 9, 1881.

Would time permit, it might not be uninteresting to scan the arguments put forth in support of these various hypotheses; that the disease is a spasm of the heart; that it is a paralysis of the heart; that it is not a disease of the heart at all, but is a neurosis, and that its essence is a hyperesthesia of the cardiac plexus; that its seat is in the system at large, and consists in an abnormal contraction of the circular fibres of the arterioles, and that it is hastened by the work of forcing the blood through these abnormally narrowed channels.

All of these theories are supported by ingenious arguments of able men, and I strongly suspect that the future will show that there is a germ of truth in each. But we must have a theory, and to my mind there is none more scientific and more practical than that adopted by Fothergill in his recent work, "The Heart and its Diseases." Still, I would caution you that it is only a theory, and that it may never see verification. Fothergill divides angina into two classes, true and false. True angina consists in an acute distension of the left side of the heart, as the result of vasomotor spasm of the arterioles, and a consequent rise in blood pressure; ordinarily it is found in cases where the arterioles have undergone fibroid degeneration from long continued lithæmia, and when at the same time the heart's fibre is materially awakened by fatty degeneration. And it is only in the presence of such organic conditions that an attack of angina becomes so serious an affair.

False angina occurs in hysterical women, and is not so distinctly connected with arteriole spasm. My own conviction is that the distinctions thus set up cannot be maintained. That the conditions of the circulation are similar in both cases; that in both there is marked high arterial tension; that in the so-called false angina a sound heart is struggling against this overburden, while in the true variety the heart is enfeebled by fatty disease, or is poorly fed by coronary arteries narrowed by atheroma. Certain it is, that hysteria is frequently associated with high arterial tension, and my observations with the sphygmograph show that in every case of sterno-cardiac pain, occurring as it so frequently does in women about the menopause, high tension would be present, and in many cases I have been able to predict the coming of an attack, days before its appearance, from a peculiarly wiry state of the pulse. I think, then, that we will do well to consider that an attack of angina pectoris is the warning of a tired heart, staggering beneath the weight of the circulation, made more burdensome by vasomotor spasm.

When we quit the field of pathology and begin the description of the symptoms of angina, the necessity for cautious speech ceases. There may be serious doubt regarding the nature or the cause of angina; there are none whatever concerning it as an assemblage of symptoms. As students we are interested in the former, but as practitioners our concern is with the latter.

Clinically considered, angina consists of an attack of pain of a peculiar character, located in the mid-sternal or precordial region, associated with this is a peculiar impression of impending death. Observation has taught me that there is great variety in this pain, and writers who delight in discovering different varieties of disease, base their classifications of the kinds of angina upon the differing characters of this pain. And there is certainly a great difference between the description of the complaint in the hysterical girl, and in the old man whose heart we have good reason to suspect is being starved from narrowed coronaries.

An attack of angina, as seen in a man of advanced years, is like nothing else in the world, and a vigorous man with nerves of steel and dauntless courage, will be reduced in the space of a few moments to the state of utter helplessness. He will hold out to you a cold, pulseless hand, and in a thin, scarcely audible voice will implore you to do something for his relief, that this pain is killing him.

All authorities agree in making prominent the essential unbearableness of the pain. Said one of my patients: "It is not pain; it is indescribable misery." Said another whom I found speechless, when relieved so as to be able to speak: "There is no honor in that pain." Another remarked, "My breast is one solid pain." There can be no doubt that in some cases the nature of the pain is most distinct, and that there is very little danger of mistaking its presence. The seat of the pain is distinctive, but subject to considerable variation. The favorite location is mid-sternal, but it may come in any part of the front of the chest, and generally has a preference for the left side. Then there is a most decided tendency to extend into the shoulders and down the arms; here the preference being shown to the left side. These extensions are referred to as radiations, and this in general is my experience; but in one of the most marked cases I have observed the pain came first in the left wrist, then in the right, slowly spreading up both arms, involving all the tissues in its course, and at length the two pains would unite over the heart. When the paroxysm yielded, the retreat was in the reverse order to the onset, the pain disappearing from the left wrist last. In the case of the late Dr. Pratt, of this city, the pain was present in the left fore-arm most of the time for years before the fatal attack.

Besides the pain, which remains by far the most prominent and persistent symptom, there are other signs which should be observed. The pulse, as you would suspect, is always changed, but in a way that requires critical examination for its detection. My observation is that the principal alteration in the pulse is in its volume. In patients with whose circulation we are reasonably familiar, we see that the pulse, instead of being full and soft, is small and resisting, and tells at once of a contracted artery. The rate per minute will not ordinarily be changed, but when changed, it will be by slowing. One of my patients whose normal pulse was 80, I found with a rate of 60 and 64 .Authorities give several peculiarities of the pulse, such as intermittent, wiry, feeble and rapid. I have never met any of these during the attack. The general condition of the patient needs a moment's consideration. In some cases we find the face white and pinched, with cold perspiration standing on the forehead, the look of the man being a blending of anxiety and terrible suffering, and we will be surprised at how fast he has grown old; sometimes he will be extremely restless, unable even to keep in bed, and we find him pacing the floor, but more often he will be fixed in one position with such terrible earnestness that shows at once his feeling, that to move a hand is to court instant death.

One thing more regarding the pulse: During the attack, as before stated, we may find the pulse regular, and neither very fast nor very slow; but after a severe attack we will be sure to find a decided change in the heart's action. It would seem as if the heart had been well nigh exhausted in the attack, and the next day we find the pulse weak, small, rapid, and frequently intermitting. I believe this phenomenon of the circulation will come to have diagnostic significance, inasmuch as intercostal neuralgia or neuralgia of the diaphragm are not accompanied with this change in the pulse. I have said nothing of the breath; but this we must observe carefully, for we will have to diagnose between angina, and asthma, and perhaps pleurisy. During the attack the breathing is rapid and shallow, but on calling our patient's attention to his lungs, as by asking him to take a full breath, we will find that he can fill the lungs fully and freely, and upon listening, we will not hear any of the sounds peculiar to any affection of the lungs.

Before leaving the signs of angina, it will be well to call attention to some of its sequlæ.

These, so far as my observation and reading go, depend upon the terribly depressing effect of an attack of angina upon the heart's power, and I will again mention that one may expect for days to find the heart's action notably weakened. As the result of this withdrawal of the vis-a-tergo of the circulation, congestions are liable to take place, preferable in the lungs, but also in the brain, the kidneys and the liver.

This complication, congestion of the lungs, was the cause of the death of my uncle, Dr. Pratt, and of my mother, and seriously threatened the lives of two cases that I can recall. As I look back upon my earlier cases, I cannot but feel that had I then known, as I now know, this tendency to lung complication of angina, I should have been upon my guard, and might have done something to ward off the attack which I then never suspected and only recognized when fully developed. I feel that I am only doing right to strongly impress this liability to fatal complication, and to urge that it be kept in mind.

This naturally brings us to the consideration of what we shall do for angina pectoris. We will remember that we have to do with an overburdened, fainting heart, and with a patient who is being killed by pain. We must be prepared to act wisely, efficiently and promply. Many times we will be too late, and find the struggle over, the patient in the rest that knows no waking; and then again, a glance will show us that there is not a moment to loose. The patient, with an old, shriveled, cold face, with scarcely perceptible respiration, and voiceless, will extend to us in mute supplication a cold, wet, pulseless hand, and with the other will indicate the seat of his trouble. Or he may be able to gasp, "You must do something to help me, quick." We have at our disposal three great agents: morphine, heat, and ammonia. We call for water, and set about getting ready a hypodermic injection of morphine. This will protect his nervous system from the deadly influence of the terrible pain, and may also be directly curative according to the theory of Balfour, which makes angina consist in a state of acute heart failure, brought about by the operation of two causes: first, the directly depressing effects of the pain—the most acute and severe the human frame can experience; and second, a condition termed sub-paralysis of the parts supplied by the motor nerves, whose sensitive roots are being thus pained. Balfour holds that this condition of sub-paralysis is the direct result of the pain, and is more or less intense, as the pain is more or less severe, and can only be relieved by first neutralizing the pain, and that this can only be done by a pure anodyne or anæsthetic, and recommends chloroform or morphia. While we are getting this ready, we order hot mustard baths for the feet and arms, hot bottles to keep up the influence when the water is removed; we order brandy, and after quickening it with aqua ammonia, will literally pour it down.

The heat, the ammonia and the brandy will each act upon the circulation, the heat and the brandy by relaxing the spasm which exists in the arterioles, thereby greatly relieving the heart's labor, and the ammonia by stimulating the heart to just one more struggle, just one, and that for life. When there is time to write a prescription we can order equal parts of Hoffman's anodyne and aromatic spirits of ammonia, or equal parts of the ammonia and compound spirits of lavender, and from one of these mixtures will add a teaspoonful to each dose of brandy. It is also well to order tr. of glonoin, and to give, of that, 5 drops in a wine glass of water, or of sling, with order to repeat every four hours.

The attention of the profession was called to the action of nitro-glycerine in angina by William Murrell, M. R. C. P., in the *London Lancet*, of 1879. The writer gives the result of sundry experiments made upon himself, and upon his professional friends, which seem to establish the fact that nitroglycerine has the same action as nitrite of amyl upon the capillary circulation, but that it differs from the amyl in that its action is much more prolonged; and that by repeating doses once in four to six hours, he could keep the circulation continuously under its peculiar influence. Upon the theory that vasomotor spasm exists as a factor of angina pectoris, a remedy which acts by relaxing this spasm suggests itself as being directly curative. All the cases I have seen published, go to show that when given in angina, nitro-glycerine acts uniformly and beneficially. I have given it in several cases of lead colic, and have found the high arterial tension peculiar to this condition to be promptly relieved, and that with this improvement the pain and constipation also disappear. In angina pectoris my experience is limited to two cases, both old men, one of 82 and the other at 78 years. In both of these cases the attacks were of the greatest severity, and the glonoin was used in connection with the other remedies and expedients before advised. I could not, as Murrell seemed to do, keep off the attacks, but my patients both recovered, and from what seemed to be the brink of the grave. I am firmly persuaded that enough is known of the action of glonoin to warrant us to make use of it in angina and in other cases where high arterial tension appears as a factor.

It has been my practice to begin its administration with 5 gtt. every 4 or 6 hours. The usual solution is of one per cent., and is said to be perfectly safe to carry or handle in any way. Before concluding this subject, I wish to say a few words upon chronic angina pectoris. My impressions may have been quite fragmentary, and not at all representing the accredited belief of the profession upon the subject, but from what I have read and heard, it did not seem to me possible that there could be such a thing as chronic angina; nevertheless such cases exist. I do not mean to be understood that I supposed that a man always died from his first attack; but my impression was that a man was entirely free from angina between the severe attacks; that the attacks might come occasionally for years before the final one; but that when the seizure came, it was sharp, severe and abrupt in its onset, and the same in its departure. Now I know that a man can have angina every day for five years or more, and not lose a month's time from business during the whole period.

That is my idea of chronic angina. The man cannot climb stairs; nor walk more than one or two blocks; nor walk against the wind; nor black his boots; nor pull them off; nor get annoyed or angry. He cannot have acid stomach, nor a flatulent stomach, nor colon, nor any form of stomach indigestion, without causing classical angina, often so severe as to threaten life. Should I

have the privilege of surviving him, I hope to be able to present the heart of such a man to the Club, with the expectation of finding some permanent reason for such a continuous and unvarying clinical history.

Theoretically, we might suspect that the walls of this heart were seriously weakened by fatty disease or starved by narrowed coronaries, and it is certain that at no time has the man any but the poorest exhibition of heart power; and yet case after case in which the autopsy shows these conditions to exist, comes to the very last hour without a single symptom pointing to trouble with the heart.

Yesterday it was my privilege to witness the autopsy of a man who suffered for over eight years with well-marked angina. His life was seriously threatened in his first attack, but being a man of the persistent and minute correctness of habit, he continued to live in the constant shadow of death for so many years.

His final illness began about eight weeks ago with a most marked and severe attack of angina, during which, in the absence of his physician, I was called upon to attend him. It was in his case that the peculiar pain, coming first in the left wrist, was observed. Aside from this there was nothing noteworthy in the attack which yielded promptly to the treatment. I learned from his physician that the attacks of pain came to be more and more frequent, and towards the close of his life were well nigh constant.

What his treatment was, I did not learn. At the autopsy in the presence of Drs. Folwell and Barker, and other members of the profession, the accompanying specimens were obtained. The heart was thought to be decidedly hyperthrophied, and upon the right side distinctly fatty. The coronary arteries which you here see, are universally atherosed and decidedly narrowed. The valves were nearly normal, as was the aorta, and there were no other evidences of arterial disease found.

But a few years since some of us attended an autopsy of a man in the same walk in life as the subject of yesterday's examnation, and of about the same age, who had complained for a few days of dyspepsia, but had not been sufficiently ill to call a physician, and who literally dropped dead instantly. I remember the case distinctly, partly from the fact that the two men lived as near neighbors in the same block. The revelations of the autopsies were almost identical, and yet nothing could be more unlike than their clinical histories. How shall we construe these seemingly discordant facts? To my mind the lesson is this: In cases of angina pectoris, not plainly hysterical, we are to conclude that the ventricular walls are seriously weakened from scanty or faulty blood supply. That in the presence of such conditions great care may prolong the life to its full term, or that death may occur at any time with scarcely a moment's warning. Surely we should have no ordinary interest in a disease where there is so much at stake, and so much to be gained by a wise and timely exhibition of the resources of our art.

